# The association between intimate partner violence type and mental health in migrant women living in Spain: findings from a cross-sectional study

**DOI:** 10.3389/fpubh.2023.1307841

**Published:** 2023-12-07

**Authors:** Abigail Bentley, Gabriel Riutort-Mayol

**Affiliations:** ^1^Instituto de Investigación en Políticas de Bienestar Social (Polibienestar), University of Valencia, Valencia, Spain; ^2^Fundación para el Fomento de la Investigación Sanitaria y Biomédica de la Comunitat Valenciana (FISABIO), Valencia, Spain

**Keywords:** intimate partner violence, depression, anxiety, migrant women, cyber IPV, economic IPV, Spain

## Abstract

**Introduction:**

The association between intimate partner violence (IPV) and mental health has been clearly established in the literature, however the differential associations between IPV type and mental health are less well understood, particularly in migrant groups who are at increased risk of both IPV and poor mental health. Under-studied and emerging forms of violence such as economic abuse and technology-facilitated abuse must be considered alongside more traditionally studied forms of IPV in order to fully understand the complex nature of violence. This study makes a novel contribution to the literature by assessing multiple forms of IPV including psychological, physical, sexual, economic and technology-facilitated IPV and their relationship with symptoms of depression and anxiety in migrant women, disaggregated by IPV type.

**Methods:**

A cross-sectional survey of migrant women living in the Valencian Community of Spain was conducted, to assess experiences of IPV and symptoms of mental health. Regression analysis from the Bayesian perspective was performed.

**Results:**

1,998 women accessed the survey. They had an average age of 37, and came predominantly from Europe (49%), namely Western Europe, followed by Latin America (38%). The majority had been in Spain between 1 and 3 years, and 80% had resident status. A total of 1,156 responded to questions on violence and mental health. Results showed that the prevalence of IPV was high, with 59% of women reporting any experience of violence. Economic abuse was the most commonly reported form of violence, and showed the strongest relationship with symptoms of depression. Sexual violence was the strongest predictor of anxiety. In both cases, in the presence of violence, the odds of having more severe symptoms of depression and anxiety increases by over 2.25. Technology-facilitated abuse was as detrimental to women’s mental health as face-to-face violence.

**Discussion:**

The findings from the study are relevant to researchers, policy-makers and service providers. They highlight the complex nature of IPV experiences faced by migrant women and the importance of understanding how different types of IPV can impact migrant mental health, in order to ensure survivors receive adequate care.

## 1 Introduction

The mental health impacts of intimate partner violence (IPV) have been investigated for a number of years, with many studies showing an association between experiences of IPV and poor mental health, including suicidal ideation, post-traumatic stress disorder, depression, anxiety, psychological distress and substance use (1–4). Studies have suggested a bidirectional association between IPV and mental health outcomes, including depressive symptoms and substance use (1, 5), with IPV demonstrated to be a predictor, as well as an outcome, of poor mental health in some longitudinal studies (6, 7). Whilst IPV can be experienced by all sexes, it predominantly affects women, with one in three women disclosing they have experienced IPV in their lifetime (8). The World Health Organisation defines IPV as ‘behaviour by an intimate partner or ex-partner that causes physical, sexual or psychological harm, including physical aggression, sexual coercion, psychological abuse and controlling behaviours’ (8). Other forms of IPV continue to emerge however, including economic violence and violence associated with the growing use of technology. In this article, the reader will find a study that quantifies the impacts of IPV on mental health in a large sample of migrant women in Spain, including the more traditionally studied forms of IPV, such as psychological, physical and sexual violence, but also emerging forms and those of more recent interest such as technology-facilitated and economic violence.

Economic abuse has been viewed as a separate and distinct form of IPV from psychological or emotional abuse only relatively recently (9–11). It has been defined as behaviours that impact a person’s ‘ability to acquire, use and maintain resources by threatening her economic security and potential for self-sufficiency’ (12), with studies conceptualising economic abuse under three constructs: economic control, economic exploitation and employment sabotage (9). The term economic abuse can include financial abuse, which is linked directly to individual money and finances (13), however economic abuse may extend to the control or restriction of wider economic resources including employment, education, transport and housing, which ultimately lead to decreased self-sufficiency and increased dependency on the abuser (9, 11). It has been difficult to fully measure or conceptualise economic abuse due to its intricate cross over with the gendered issue of economic insecurity that characterises women’s experience in general (9).

The use of technology to facilitate IPV could include using text, email or social media to harass or abuse a partner, using apps or stalkerware to track their whereabouts and monitor online activity, and the remote use of cameras, microphones and smart home devices to spy on and manipulate the environment, thereby isolating, punishing or humiliating a partner or creating feelings of being constantly under surveillance (2, 14–16). It has been estimated that almost half of people who experience IPV will experience some form of technology-facilitated abuse (17), which has been defined under three main constructs: psychological, involving emotional harassment and abuse via devices and social media; sexual, including forced or coercive sharing or receiving of sexual content; and stalking, including monitoring accounts, devices and whereabouts using technology (18). Although limited studies exist, some researchers have shown that technology-facilitated abuse is associated with symptoms of poor mental health, including depression, anxiety, post-traumatic stress disorder and substance use (19–22).

White et al. (4) highlighted the importance of disaggregating analyses by type of IPV when assessing the association with symptoms of mental health in order to understand the impact of different types of violence (4). Few studies have looked at the association between differential exposures to IPV and mental health, however of those that have, there have been mixed findings. A meta-analysis of global studies found that sexual IPV showed the strongest association with distress compared to physical and psychological IPV (4), and a review of studies on intimate partner sexual violence found that it was more strongly related to post-traumatic stress disorder, depression, substance use and suicidality than experiences of non-sexual IPV (23). However, other studies have shown that psychological violence has a stronger relationship with symptoms of poor mental health, particularly depression, than physical or sexual violence (24–26). A meta-analysis of studies on psychological violence demonstrated that this type of IPV overall was strongly associated with symptoms of depression, anxiety and post-traumatic stress disorder. Emotional/verbal abuse and dominance/isolation showed a stronger association with symptoms of depression than coercive control, whereas all forms of psychological abuse were strongly associated with post-traumatic stress symptoms (27). The majority of studies assessing the differential association between IPV types and mental health have looked only at psychological, physical and sexual violence, and have not taken into account newer forms of violence such as economic violence and technology-facilitated abuse. One review of studies on economic violence found that it was associated with depression, anxiety, psychological distress and suicidal attempts (11), and some studies have found that economic abuse was more strongly associated with symptoms of depression than other forms of violence (10, 28). Technology facilitated abuse—or cyber IPV—has also been associated with mental health sequelae (20, 29, 30).

Migrant women are at increased risk of experiencing IPV for numerous reasons, including often being isolated with limited family and social support, having limited access to economic resources, facing barriers to accessing services, and legal restrictions due to undocumented or non-permanent immigrant status. This can make them increasingly vulnerable, particularly if threatened with being reported to child protective services or immigration authorities, as can often happen in abusive situations. These individual-level factors are also often rooted within a cultural context of traditional gender norms, acceptance of violence against women and a high stress environment linked to acculturation difficulties, downward mobility and discrimination that can increase the risk of IPV (31, 32).

In addition, migrants are at increased risk of having poor mental health. Factors experienced pre-migration, during migration or post-migration, including the disruption and loss of family and social support networks, exposure to harsh living conditions, violence and trauma, unemployment, loss of social status, uncertainty about immigration or refugee status and acculturation difficulties have all been associated with poor mental health in migrant groups (33–35). Structural violence experienced in the destination country can also impact the mental health of migrants (36). Migrant women face additional risk factors to their mental health, including institutional, social and internalised discrimination, exposure to male violence, including IPV, at all stages of the migration journey, as well as elevated stress linked to the unequal burden of caring responsibilities, and fewer opportunities for work and socialisation due to gender and cultural norms (37).

Research has shown that there is an association between IPV and poor mental health in migrant groups. A longitudinal study in Australia comparing refugee women to Australian-born women in antenatal settings found that not only did refugee women have a significantly higher prevalence of IPV, but they also had a higher prevalence of major depressive disorder (31). IPV in South Asian immigrant communities has been linked to elevated levels of poor mental health including depression, suicidal thoughts and suicidal attempts (38), and a qualitative study with Latin American women in Canada highlighted a strong relationship between IPV and poor mental health, including depression (39). Socially marginalised women who are exposed to IPV are also at an increased risk of suicide (4). Whilst studies are limited, the differential impact of exposure to different IPV types on mental health has been demonstrated in some migrant groups (40–42).

As of July 2023, it is estimated that there are almost 3,200,000 migrant women living in Spain (43). Studies have shown that the prevalence of IPV in migrant women is higher than that of Spanish born women, including for physical and psychological IPV (44–47). To our knowledge, there have only been two studies to date that have assessed both IPV and mental health in migrant women in Spain. Both found an increased risk of poor mental health in women who had experienced IPV, however they included only physical, psychological and sexual IPV and did not fully disaggregate by type of violence when investigating the association with mental health (44, 46). The Valencian Community has one of the largest migrant populations in Spain behind Catalonia and Madrid with almost 800,000 registered foreigners in 2022, and it has seen the largest increase (18%) in its migrant population of these three major Spanish hubs since 2018 (48). In 2021, the Valencian government released their Valencian Strategy on Migration, which included a goal to ensure that they meet the needs of migrant women, specifically, ‘implementing the measures provided for in the Valencian Pact to Fight Gender-based Violence and Male Violence against Women for the integral specialised care of migrant women, taking into account their greater vulnerability’ (49). However, in order to achieve this goal, it is important to first understand the experiences of such migrant women living in the region.

The aim of this study was to fill a gap in the literature by investigating the impact of different forms of IPV on the mental health of migrant women living in the Valencian Community of Spain. We had two main research questions: (1) what are the patterns of psychological, physical, sexual, economic and technology-facilitated violence experienced by migrant women living in Valencia? (2) What is the association between each of these forms of IPV and symptoms of depression and anxiety? The novelty of this work lies in the inclusion of economic and technology-facilitated abuse alongside more traditionally studied forms of IPV, the disaggregation of IPV type in the assessment of the relationship between violence and mental health, as well as the study of migrant women in Spain.

## 2 Methods

### 2.1 Data collection

Data were collected via an online cross-sectional survey of migrant women living in the Valencian Community of Spain using social media sampling through the use of targeted adverts posted on social media platforms, including Facebook and Instagram, to recruit participants. With the rising use of social networking sites, social media sampling has been increasingly used in research studies to access marginalised and hard-to-reach populations where probabilistic sampling methods are challenging, because it allows for highly specific targeting, offers greater anonymity to vulnerable populations, reduces barriers to accessing research studies and has been cited as more effective than other more traditional methods in terms of time, cost, and numbers of participants recruited (50, 51). International migrants are considered to be a particularly hard-to-reach population to engage in research through traditional methods due to high mobility, socioeconomic situations, distrust in institutions, language and cultural barriers and reluctance to engage with researchers due to fears of negative impacts on their legal status (52). Given that we wanted to target migrant women in our study, we opted to use social media sampling as it provided us with a viable option to reduce some of these barriers and reach a larger number of our target population, across a wide geographical area. Adverts were targeted at women aged 18 and above who were living anywhere in the Valencian Community and were run in Spanish, English and French. Respondents were offered the opportunity to enter into a prize draw to win a €90 voucher for El Corte Ingles for participating in the study. They were not required to complete the full survey or answer any questions that they did not want to in order to be entered, but they must have started the survey and indicated that they wanted to be entered in order to participate in the prize draw.

On accessing the survey, respondents were presented with four initial questions to assess their eligibility to participate and ensure that the population of interest was captured. These questions required that respondents be non-Spanish women over the age of 18 currently living in the Valencian Community. Once respondents passed the eligibility questions, they were taken to a landing page that included the full participant information sheet outlining the details of the study, what they could expect, and how their data would be treated and stored. Informed consent was taken before participants could proceed to the full survey. The survey was available in Spanish, English and French and participants could select their preferred language before beginning. 1,998 women accessed the survey and consented to take part. Forty-five percent chose to complete the survey in English, 44% in Spanish and 11% in French.

### 2.2 Socio-demographic information

The survey collected socio-demographic information about participants including their age, country of origin, ethnicity, education level, sexual orientation, whether they had ever been married or lived with a partner, whether they had children, how many and their ages, whether they currently worked, which included a response option ‘not permitted to work’, their approximate monthly household income, who they currently lived with, whether they had resident status, how long they had lived in Spain and whether they felt they had a network of social support in Spain.

### 2.3 Mental health variables

#### 2.3.1 PHQ-9

The PHQ-9 is the 9-item depression module of the wider Patient Health Questionnaire, a self-report questionnaire developed in primary care settings in the United States in the late 1990s to measure ‘mental disorders’ (53). The module includes 9 questions that ask about distinct symptoms of depression experienced in the preceding 2 weeks, and in the original study it displayed excellent reliability and validity (54). It has been validated in a variety of geographical settings, including low and middle-income countries, across clinical and community settings and across a variety of ethnic, cultural and linguistic groups (55, 56). Studies have shown that the PHQ-9 performs as well as, if not better, than other measures of depression and meta-analyses have shown that it has an adequate sensitivity and specificity to detect major depression (57, 58). It has been validated in multiple languages, including Spanish (59–61) and French (62, 63). Questions are measured on a 4-point likert scale with the response options ‘Not at all’, ‘Several days’, ‘More than half the days’ and ‘Nearly every day’, scored from 0 to 3, respectively. A total score can be calculated, with a maximum value of 27, and scores with a cut-off point of 5, 10, 15 and 20 can be categorised as ‘mild’, ‘moderate’, ‘moderately severe’ and ‘severe’ depression, respectively (57).

#### 2.3.2 GAD-7

The GAD-7 is a 7-item survey tool to measure symptoms of Generalised Anxiety Disorder experienced in the previous 2 weeks. Similarly to the PHQ-9, it was originally developed in clinical populations in the United States, where it displayed excellent reliability and validity characteristics (64). It has since been validated in multiple populations and in multiple languages including Spanish (65, 66) and French (67, 68). Response options are also measured on a 4-point likert scale from 0 to 3 of ‘Not at all’, ‘Several days’, ‘More than half the days’, and ‘Nearly every day’, with a maximum score of 21. Scores can be categorised as ‘mild’, ‘moderate’, and ‘severe’ anxiety, represented by a cut-off of 5, 10, and 15, respectively (57).

### 2.4 Intimate partner violence variables

#### 2.4.1 Revised composite abuse scale-short form

The revised composite abuse scale-short form (CASR-SF) was originally developed by Ford-Gilboe and colleagues in 2016 with a mixture of community-based and clinical populations in Canada. Based on the Composite Abuse Scale (69), the authors aimed to develop a shortened self-report measure of IPV that captured ‘the complexity of IPV, including severity, whilst limiting participant burden and enhancing emotional safety’ (70). The 15-item version consists of questions measuring a range of experiences of intimate partner violence in the past 12 months. Thirteen of the 15 items loaded onto three factors, representing three constructs within the respective subscales: psychological, physical and sexual violence. The psychological subscale consists of six questions including experiences of verbal abuse, e.g., ‘told me I was crazy, stupid or not good enough’, harassment, stalking, and controlling behaviours, e.g., ‘tried to keep me from seeing or talking to my friends or family’. The physical subscale included five items of physical violence ranging from ‘shook, pushed, grabbed or threw me’ to ‘choked me’, and the sexual violence subscale included two items related to forced intercourse and sexual acts. The two remaining items were ‘threatened to harm or kill me or someone close to me’ and ‘kept me from having access to a job, money or financial resources’. Experiences of each act of violence are first measured with a binary ‘yes’ or no’ response. If a respondent selects ‘yes’ to an experience of violence, a follow up question is asked to measure the frequency of the experience in the past 12 months. Frequency is measured on a 6-point likert scale from 0 to 5, ‘Not in the past 12 months’, ‘Once’, ‘A few times’, ‘Monthly’, ‘Weekly’, ‘Daily/almost daily’ (70).

#### 2.4.2 Revised scale of economic abuse

The revised scale of economic abuse (SEA2) is a 14-item scale developed by Adams et al. (71) to measure economic abuse within intimate partnerships. It was validated with adult women accessing domestic violence services in the United States. The scale measures the presence and frequency of acts of economic abuse experienced in the past 12 months, measured on a 5-point likert scale ranging from ‘Never’ (0) to ‘Very often’ (4). The items load onto one of two subscales. The first relates to ‘economic restriction’, which describes how perpetrators limit their partners’ access to and use of economic resources. It includes questions such as ‘Decide how you could spend your money rather than letting you spend it how you saw fit’, ‘Make you ask him or her for money’, and ‘Keep you from having a job or going to work’. The second subscale, ‘economic exploitation’, involves ‘forcibly, coercively, or fraudulently using a partners’ economic resources for one’s own advantage’. It includes acts of abuse such as ‘steal your property’, ‘force or pressure you to give him/her your savings or other assets’, and ‘take out a loan or buy something on credit in your name without your permission’ (71).

#### 2.4.3 Cyber aggression in relationships scale

The CARS was developed by Watkins et al. (18) to assess the presence of cyber abuse, or technology-based violence, within intimate partnerships. The scale was developed with an online sample of men and women in the United States and consists of 34 items, 17 of which measure victimisation and 17 of which measure perpetration. For the purposes of this study, only the victimisation questions were used. The scale has a three-factor structure relating to three subscales of cyber abuse: psychological, sexual and stalking. Psychological cyber abuse refers to using technology-based information such as photos, videos or messages to cause harm. This could be to insult, threaten, harass or humiliate a partner either in public or private, such as posting humiliating content on social media, or sending threatening messages via text or social media. Sexual cyber abuse includes pressuring or coercing partners into engaging with unwanted sexual acts, and posting, sending or receiving unwanted sexual content via technology. Stalking cyber abuse refers to the monitoring and surveillance of partners through the use of technology, including accessing accounts and devices without permission, tracking whereabouts through GPS, and monitoring internet activity. Items are measured on an 8-point Likert scale, with response options including ‘never’, ‘not in the past 6 months but it did happen before’ and then ranging from ‘Once in the past 6 months’ to ‘More than 20 times in the past 6 months’ (18).

Permission was sought from authors of all violence measurement scales prior to their use in this study. Socio-demographic and violence questions were translated from English into Spanish and French using forward and back translation with professional translators.

### 2.5 Data management

#### 2.5.1 Socio-demographic variables

Socio-demographic variable categories were collapsed where necessary for the regression analysis to reduce the chances of complete separation in the data due to small numbers. Country of origin was summarised into regions including ‘Europe’, ‘Africa, Asia, and the Middle East’, ‘Latin America’ and ‘North America/Other’. ‘Other’ in this context included respondents from Australasia or from multiple countries. Ethnicity was collapsed into categories that included ‘White’, ‘Latina’, ‘Central Asian, Middle Eastern or North African’, ‘South, East or South-East Asian’, ‘Black’ which represented women who identified as Black European, Black American, Black African, Black Latina and Black Caribbean, and ‘Other’ which included women with multiple ethnic identities. Similarly, religion was reduced to ‘No religion/atheist’, ‘Christian/Catholic’ and ‘Other’. As all respondents reported that they had completed secondary education, education level was summarised as ‘Secondary education’ or ‘Higher education’ and monthly household income was categorised as ‘low’ (<€1,000), ‘medium’ (€1,000 to €3,000) and ‘high’ (€3,000+).

#### 2.5.2 Mental health variables

Given that the discrete scores on the PHQ-9 and GAD-7 were upper-bound truncated random variables, meaning that scores only exist within a set range and have a maximum value that is greater than or equal to all other values, and in order to improve and simplify the interpretability, we decided to use categorised versions of these variables in the analysis. In line with the scale recommendations, PHQ-9 scores with a cut-off point of 5, 10, 15, and 20 were categorised as ‘minimal’, ‘mild’, ‘moderate’, ‘moderately severe’, and ‘severe’ depression, respectively. The GAD-7 scores were categorised as ‘minimal’, ‘mild’, ‘moderate’ and ‘severe’ anxiety, represented by a cut-off of 5, 10, and 15, respectively (57).

#### 2.5.3 IPV variables

Given that for this study we were interested in the association between the presence of violence experiences and mental health outcomes, we created dummy variables to summarise the IPV scales. For each scale, CASR-SF, CARS and SEA2, a global binary summary variable (yes/no) was created to indicate the presence or absence of any act of violence, in addition to a binary variable to indicate the presence of violence within each subscale: psychological, physical and sexual violence within the CASR-SF; psychological, sexual and stalking violence in the CARS; and economic exploitation and restriction in the SEA2. Furthermore, a variable was created to indicate the number of different types of violence experienced, based on all available subscales measuring distinct different types of violence. The maximum number was 8 and included all of the subscales mentioned above. Finally, another variable was created to calculate the number of distinct different acts of violence experienced, counting the total number of violence items across all scales with a positive response. The maximum possible response was 46. These two latter variables, the number of different types of violence and the number of different acts of violence, were to serve as a proxy for polyvictimisation and severity, respectively, with a higher number of acts of violence and a higher number of different types of violence suggesting the presence of complex violence patterns often experienced by survivors (72). A total of 13 independent variables were therefore generated to assess experiences of violence in the sample population, which have been summarised in Table 1.

**Table 1:** Derived variables from each violence scale and subscale.

CASR-SF	Global (any experience)	Binary (yes/no)
	Psychological	Binary (yes/no)
	Physical	Binary (yes/no)
	Sexual	Binary (yes/no)
SEA2	Global (any experience)	Binary (yes/no)
	Economic restriction	Binary (yes/no)
	Economic exploitation	Binary (yes/no)
CARS	Global (any experience)	Binary (yes/no)
	Psychological	Binary (yes/no)
	Stalking	Binary (yes/no)
	Sexual	Binary (yes/no)
Number of distinct different types of violence		Count 0 to 8
Number of distinct different acts of violence		Count 0 to 46

### 2.6 Data analysis

A descriptive analysis was performed to assess the distribution and variability within the data. Measures of spread including the range, mean, standard deviation, median and interquartile range were used to assess continuous variables. Frequency and proportion distributions were used to assess categorical variables. Histograms and box-plots were used to visualise the data and identify potential outliers.

To take an initial look at the relationship between socio-demographic and mental health variables, a multivariate regression analysis was carried out and the resulting associations evaluated. We did the same to assess the relationship between socio-demographic and IPV variables. Socio-demographic variables that displayed an association with mental health and IPV variables were selected as confounders in the multivariable regression analysis to evaluate the association between IPV and mental health. We had two variables to represent whether or not women had children, one of which indicated children in general, and another that indicated whether women had children aged over 18. We chose to use the latter in the subsequent analyses because whilst at the univariable level, both variables had a similar relationship with the violence variables, the child >18 variable had a stronger association with the mental health variables and was the only one that remained statistically significant at the multivariable level. Similarly, as there was a high level of correlation between the ethnicity and region of origin variables, we chose to retain only one of these. The final covariates included in the model were: age, whether or not the woman had a child aged 18 or over (‘child > 18’), whether the woman had ever been married or lived with a partner (‘Ever married’), the approximate monthly household income (‘Income’), the woman’s current living arrangements (‘Liv. arrange’), the woman’s region of origin, her religion, whether or not she had resident status, whether or not she perceived she had social support, her length of time in Spain and her working status.

The regression analysis to evaluate the associations between IPV and mental health variables consisted of an ordinal logistic regression, formulated from a Bayesian perspective and adjusted using sampling methods in the probabilistic programming software Stan (73, 74). In ordinal logistic regression, the value of a coefficient associated with an independent continuous or categorical variable is presented as an odds ratio, which represents the variation of the logarithm of the odds between categories. The exponential of the coefficient represents the variation in odds between categories. The odds are the ratio between the probabilities that the observed response is greater than or equal to and less than a certain category k_j,


Ρ(y_i≥k_j)∕Ρ(y_i<k_j)

where y_i is the observed response category of individual i to the ordinal categorical outcome variable with K categories;


$$y\_i=\left\{k_{j}:j=1,\;\ldots \;,K;k\_\left(j-1\right)<k\_j\right\}$$

Therefore, the exponential of the coefficient represents the change in the proportion between the probabilities of the categories of the dependent variable when the independent variable increases by one unit; a unit in a continuous variable, or a category in a categorical variable. Values of the exponential coefficient greater than 1 mean an increase in the odds, and values less than 1 mean a reduction in the odds. A value equal to 1 means no change in the odds.

Stan software uses the Hamiltonian Monte Carlo sampling method (75) to estimate the marginal posterior probability distributions of every parameter of interest. Four simulation chains with different initial values were launched. The convergence of the simulation chains was evaluated by the split-Rhat convergence diagnosis and the effective sample size of the chains (76, 77). In our case and for both models, a split-Rhat value lower than 1.05 has been obtained for all parameter simulation chains indicating good mixing of simulated chains. The convergence of the simulation chains indicates that the samples do come from the posterior distribution, and that the model is correctly specified without confusion or identifiability problems between parameters.

Magnitude and uncertainty of the parameters of interest, the odds ratios for all levels of covariates, are given by their marginal posterior distributions. Significance of the odds ratio can be determined by evaluating whether their accumulated probabilities of being less than or greater than one are lower than a probability threshold. In this study we set the commonly used probability threshold of 0.05.

The pre-processing of the data before sending it to Stan to perform model inference, as well as its post processing, was carried out using R software for data analysis and processing (78).

## 3 Results

There were 1,998 migrant women who accessed the survey in total. Of these, 1,676 (83.9%) responded to the questions on mental health and 1,312 (65.7%) responded to some questions on violence: 1,277 (63.9%) responded to the questions on physical, sexual or psychological violence (CASR-SF), 1,263 (63.2%) responded to the questions on economic violence (SEA2) and 1,211 (60.6%) responded to the questions on cyber IPV (CARS). All who responded to some questions on violence also responded to the questions on mental health. 1,156 women (57.9%) completed all questions related to mental health across the PHQ-9 and GAD-7 and questions related to experiences of violence across the CASR-SF, SEA2 and CARS. Given that our study was interested in the association between experiences of violence and symptoms of mental health, the findings of our regression analysis are based on these 1,156 respondents.

### 3.1 Socio-demographic distribution

The distribution of respondents by demographic characteristics (Table 2) is relatively similar between those who responded to the mental health questions (*n* = 1,676) and those who responded to all violence questions (*n* = 1,156).

**Table 2:** Socio-demographic characteristics of the sample migrant women, for those that responded to the questions on mental health (*n* = 1,676) and those that responded to all questions on violence (*n* = 1,156).

	Women responding to mental health questions *n* = 1,676			Women responding to violence questions *n* = 1,156		
Variable	*n*	*n*%	Mean (±SD)	*n*	*n*%	Mean (±SD)
Age	1,669	99.6%	37 (14)	1,154	99.8%	37 (13)
Missing	7	0.4%			0.2%	
**Time in Spain**						
1–3 years	418	25%		291	25%	
10+ years	325	19%		260	23%	
3–5 years	315	19%		243	21%	
5–10 years	224	13%		168	15%	
Less than 1 year	389	23%		191	17%	
Missing	5	0.3%		3	0.3%	
**Region of origin**						
Africa, Asia, Middle East	75	4%		54	5%	
Europe	804	48%		562	49%	
Western	597	74%		432	77%	
Eastern/Southern	206	26%		130	23%	
Latin America	655	39%		442	38%	
North America/Other	137	8%		97	8%	
Missing	5	0.3%		1	0.1%	
**Working status**						
Not working	679	41%		420	36%	
Not permitted to work	168	10%		103	9%	
Working	821	49%		629	55%	
Missing	8	0.5%		4	0.3%	
Alone	216	13%		116	10%	
With friends/flatmates/host	380	23%		205	18%	
With parents/other family	109	7%		67	6%	
With partner/children/both	896	54%		715	63%	
Other	53	3%		37	3%	
Missing	22	1.3%		16	1.4%	
**Ever married or lived with a partner**						
No	715	43%		367	32%	
Yes	951	57%		782	68%	
Missing	10	0.6%		7	0.6%	
**Children**						
No	1,047	63%		714	62%	
Yes	623	37%		440	38%	
Missing	6	0.4%		2	0.2%	
**Child aged 18 plus**						
No	1,380	82%		972	84%	
Yes	296	18%		184	16%	
Missing	0	0.0%		0	0.0%	
**Religion**						
Christian/Catholic	767	46%		503	44%	
No religion/atheist	802	48%		588	51%	
Other	105	6%		64	6%	
Missing	2	0.1%		1	0.1%	
**Income**						
High (€3,000+)	266	16%		198	17%	
Medium (€1,000–€3,000)	773	47%		575	50%	
Low (<€1,000)	620	37%		374	33%	
Missing	17	1.0%		9	0.8%	
**Social support**						
No	708	42%		474	41%	
Yes	962	58%		678	59%	
Missing	6	0.4%		4	0.3%	
**Resident**						
No	387	23%		227	20%	
Yes	1,283	77%		927	80%	
Missing	6	0.4%		2	0.2%	
**Ethnicity**						
Black	37	2%		26	2%	
Latina	470	31%		327	31%	
MENA/Central Asian	40	3%		23	2%	
South, East, South-East Asian	41	3%		29	3%	
White	841	56%		598	56%	
Other	85	6%		56	5%	
Missing	162	10%		97	8%	

Women who provided data on mental health ranged in age from 18 to 84, with an average age of 37 (SD 14). Almost half of respondents came from Europe (48%), with the majority from Western Europe, followed by Latin America (39%). Over half of women were White (56%), 31% Latina and 14% identified as Black, Middle Eastern or North African, Asian or Other. 10% of women did not disclose their ethnicity. 48% of women were Atheist or had no religion and 46% were Christian or Catholic.

Most women (57%) had ever been married or lived with a partner, and a similar proportion (54%) currently lived with their partner and/or children. 23% lived with friends or flatmates and 13% lived alone. Around 40% of women had children.

The majority of women (25%) had been in Spain for between one to 3 years and 77% had resident status in Spain. Half of women were currently working, with 10% not permitted to work. Half of women had an approximate household income that fell within the ‘medium’ range of €1,000–€3,000, and 58% of women felt that they had a network of social support in Spain.

### 3.2 Symptoms of depression and anxiety: description and associations with socio-demographic factors

**Figure 1:**
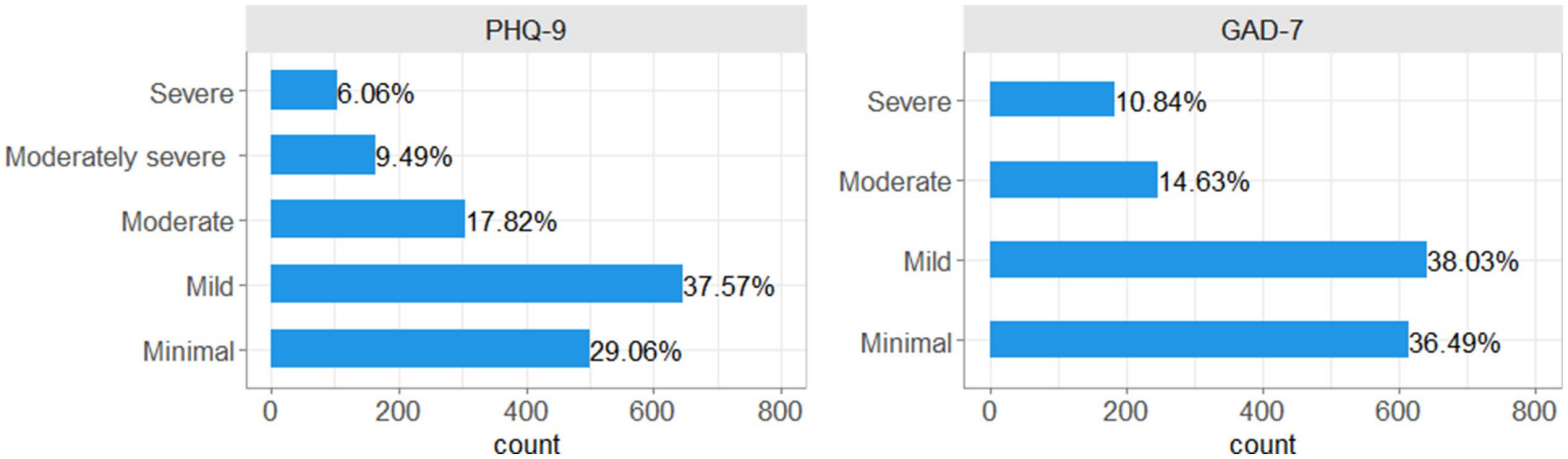
Frequency and proportion of women falling into each category of PHQ-9 and GAD-7 scores.

The majority of women (37.6%) who responded to the questions on mental health displayed symptoms of mild depression in the previous 2 weeks. However, 18% showed symptoms of moderate depression and 16% showed symptoms of moderately severe to severe depression (Figure 1, PHQ-9).

Almost 40% of women displayed symptoms of mild anxiety. However, 15% had experienced symptoms of moderate anxiety, and 11% severe anxiety in the previous 2 weeks (Figure 1, GAD-7).

**Figure 2:**
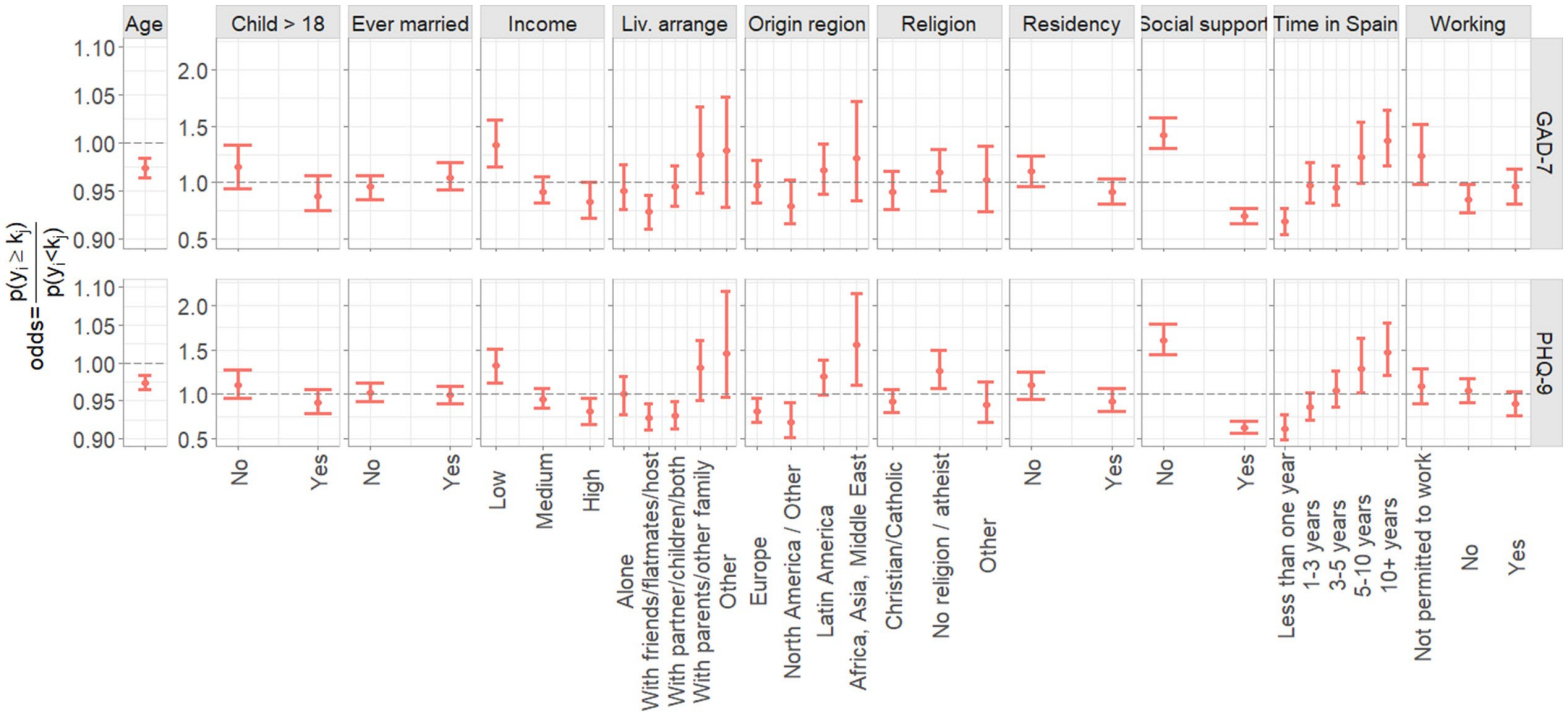
Association of socio-demographic predictors with PHQ-9 and GAD-7 scores. The age predictor effect represents the change in odds for a 1-year increase in age.

In Figure 2, the associations in terms of odds ratio between socio-demographic factors and symptoms of depression and anxiety (PHQ-9 and GAD-7 variables, respectively), are depicted. Women’s age was slightly negatively associated with symptoms of both anxiety and depression, with older women having lower odds of having more severe symptoms at the rate of over 0.97 per year of age. The longer women had spent in Spain, the more likely they were to have more severe symptoms of anxiety and depression. For example, women who have been in Spain for more than 10 years have approximately twice the odds of suffering more severe symptoms of anxiety and depression than women who have been in Spain for less than a year and approximately 1.5 times the odds than women who have been in Spain for 1–3 years. Increased income was protective against more severe symptoms of anxiety and depression, with women in the lowest income group having approximately 1.5 times greater odds of having poorer mental health than women in the highest income group. Similarly, having perceived social support within Spain was highly protective against both anxiety and depression with approximately twice the odds of having more severe symptoms for those who did not have social support. Women who came from Africa, Asia and the Middle East, or from Latin America, were more likely to have worse symptoms of mental health than women from Europe or North America/Other. For example, women from Africa, Asia or the Middle East had almost 2 times the odds of suffering more severe symptoms of depression than women who came from North America/Other. In addition, women who were not religious were more likely to have worse symptoms of depression than women who were religious (Christian/Catholic or ‘other’ religion; Figure 2).

### 3.3 Experiences of violence (IPV variables): description

**Figure 3:**
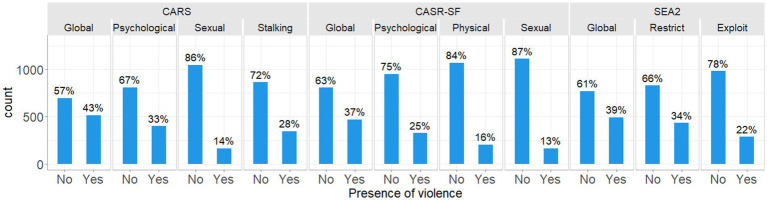
Frequency and proportion of women experiencing each type of violence as measured by the CASR-SF, SEA2, and CARS.

Of women who responded to the questions on violence, 59% had experienced at least one act of violence assessed within the survey. 37% of women had experienced at least one act of violence measured through the CASR-SF, with 25% having experienced psychological violence, 16% physical and 13% sexual (Figure 3, CASR-SF). Similar levels of technology-facilitated psychological abuse and sexual abuse were experienced as with face-to-face abuse, with 33% psychological and 14% sexual cyber abuse (Figure 3, CARS). 28% of women experienced technology-facilitated stalking and 39% of women experienced economic abuse, the most prevalent type of violence (Figure 3, SEA2).

**Figure 4:**
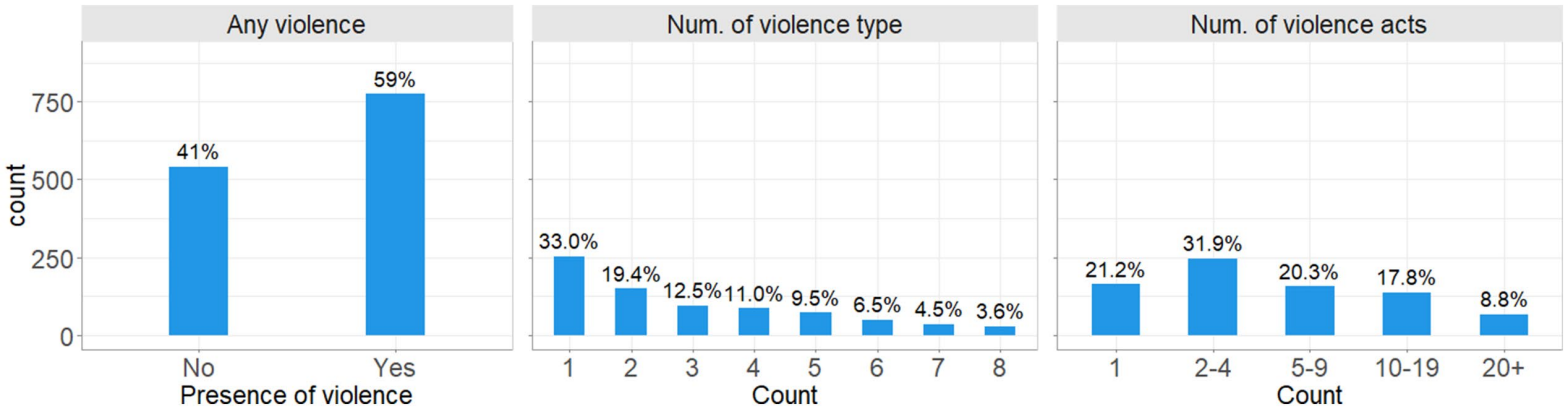
Frequency and proportion of women experiencing any violence, the number of different types of violence, and the number of different acts of violence.

Of women who experienced any act of violence (Figure 4, Any violence), the total number of different acts of violence experienced ranged from 1 to 44, with 21% reporting only one act of violence, 32% reporting 2–4 different acts, 20 and 18% reporting 5–9 and 10–19 different acts, respectively, and 9% reporting 20 or more different acts of violence (Figure 4, Num. of violence acts). When considering how these acts were split over the number of different types of violence, 67% of women experienced some form of polyvictimisation, with 19% experiencing two different types of violence, and 4% experiencing all 8 different types of violence measured, as related to the subscales of the CASR-SF, SEA2 and CARS. Around a third of women did not report polyvictimisation, with only one type of violence reported (Figure 4, Num. of violence type).

### 3.4 Relationship between intimate partner violence and mental health

**Figure 5:**
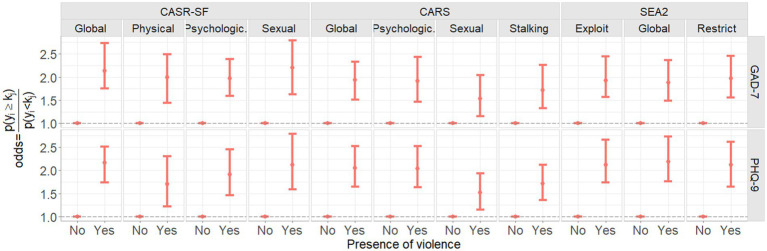
Mean and 95th percentiles (95% credible intervals) of the estimated odds ratio between CASR-SF, SEA2 and CARS global variables and subscales and PHQ-9 and GAD-7 scores.

**Figure 6:**
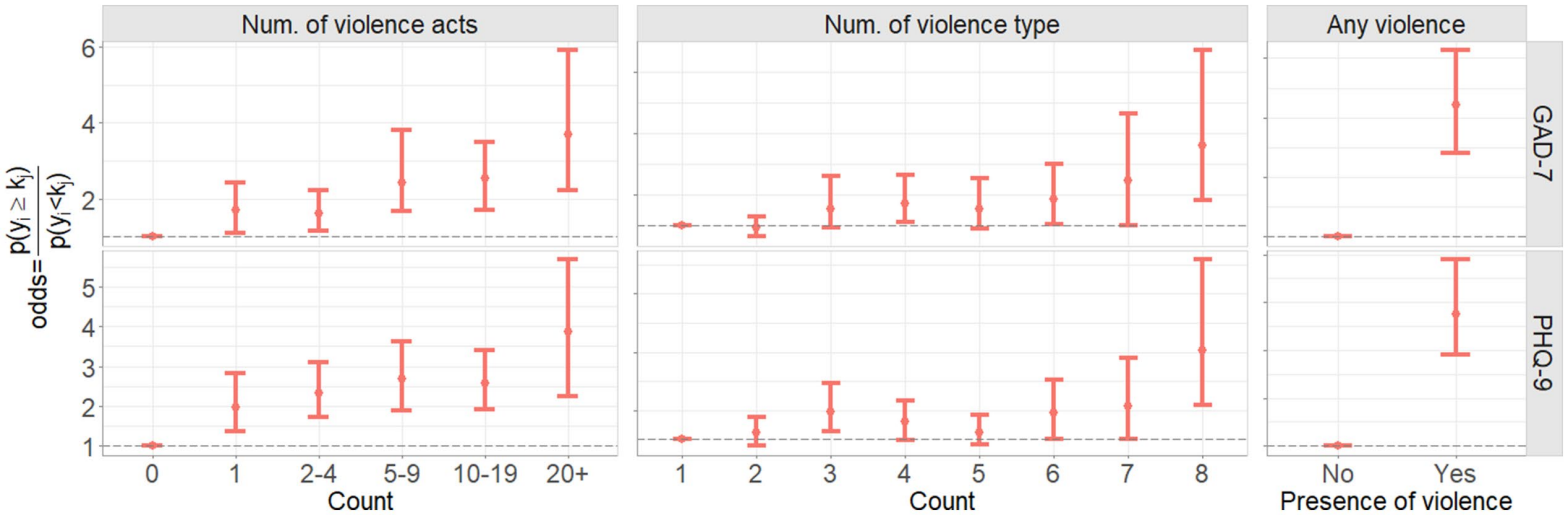
Mean and 95th percentiles (95% credible intervals) of the estimated odds ratio between the presence of any violence, number of acts of violence experienced and number of types of violence experienced, and PHQ-9 and GAD-7 scores.

Figure 5 and Table 3 show the associations in terms of odds ratio between IPV variables (CASR-SF, CARS and SEA2 scales) and symptoms of depression (PHQ-9) and anxiety (GAD-7). Furthermore, Figure 6 and Table 4 show the odds ratio between the derived ‘any violence’, ‘number of different violence type’ and ‘number of different violence acts’ variables. In the figures and tables, 95% credible intervals, and the statistical significance of the parameters by means of the probability of being different to one are presented.

**Table 3:** Association of CASR-SF, CARS and SEA2 IPV variables with PHQ-9 and GAD-7 scores: Mean of the estimated odds ratio (OR), 95th percentile (95% credible intervals) and statistical significance (P).

		PHQ-9		GAD-7	
		OR (95% CI)	*P* ^*^	OR (95% CI)	*P* ^*^
CASR-SF	Global	2.17 (1.71–2.66)	0.00	2.16 (1.72–2.60)	0.00
	Psychological	1.92 (1.50–2.36)	0.00	1.96 (1.52–2.47)	0.00
	Physical	1.78 (1.27–2.30)	0.00	2.07 (1.55–2.79)	0.00
	Sexual	2.12 (1.47–2.78)	0.00	2.22 (1.55–2.90)	0.00
SEA2	Global	2.23 (1.80–2.75)	0.00	1.89 (1.52–2.37)	0.00
	Economic restriction	2.16 (1.67–2.60)	0.00	2.00 (1.60–2.42)	0.00
	Economic exploitation	2.14 (1.69–2.72)	0.00	1.94 (1.52–2.45)	0.00
CARS	Global	2.04 (1.63–2.40)	0.00	2.00 (1.56–2.45)	0.00
	Psychological	2.01 (1.56–2.50)	0.00	1.94 (1.56–2.55)	0.00
	Stalking	1.70 (1.34–2.14)	0.00	1.78 (1.43–2.14)	0.00
	Sexual	1.54 (1.07–2.11)	0.01	1.49 (1.14–2.02)	0.00

P^*^: Probabilities of being greater or lower than 1. In a Bayesian framework, an odds ratio estimate is said to be statistically significantly increasing or decreasing if its probabilities of being greater or lower than 1 are smaller than a threshold ε, e.g., ε = 0.05.

**Table 4:** Association of derived ‘Number of different violence types’, ‘Number of different violence acts’, and ‘Any violence’ variables from IPV variables with PHQ-9 and GAD-7 scores: Mean of the estimated odds ratio (OR), 95th percentile (95% credible intervals) and statistical significance (P).

		PHQ-9		GAD-7	
		OR (95% CI)	*P* ^*^	OR (95% CI)	*P* ^*^
Number of distinct types of violence	1 (Reference category)	-	-	-	-
	2	1.20 (0.82–1.73)	0.21	0.92 (0.61–1.39)	0.68
	3	1.92 (1.17–2.88)	0.01	1.58 (1.03–2.31)	0.01
	4	1.64 (1.03–2.53)	0.01	1.63 (1.08–2.53)	0.03
	5	1.22 (0.68–2.00)	0.28	1.59 (0.98–2.41)	0.02
	6	1.90 (1.11–3.08)	0.01	1.80 (1.02–3.05)	0.03
	7	2.07 (1.08–3.51)	0.02	2.53 (1.18–4.36)	0.02
	8	3.97 (1.76–7.47)	0.00	3.75 (1.63–7.66)	0.01
Number of distinct acts of violence	0 (Reference category)	-	-	-	-
	1	1.89 (1.24–2.83)	0.00	1.69 (1.14–2.51)	0.00
	2–4	2.22 (1.56–3.08)	0.00	1.64 (1.19–2.06)	0.00
	5–9	2.62 (1.79–4.11)	0.00	2.36 (1.68–3.18)	0.00
	10–19	2.51 (1.67–3.60)	0.00	2.51 (1.70–3.56)	0.00
	20+	3.78 (2.08–6.06)	0.00	3.53 (2.18–6.00)	0.00
Any violence		2.44 (1.94–2.99)	0.00	2.12 (1.72–2.60)	0.00

P^*^: Probabilities of being greater or lower than 1. In a Bayesian framework, an odds ratio estimate is said to be statistically significantly increasing or decreasing if its probabilities of being greater or lower than 1 are smaller than a threshold ε, e.g., ε = 0.05.

Women who experienced at least one act of any type of violence had over twice the odds of having more severe symptoms of depression and anxiety than women who did not experience any violence. This was mirrored through the global score on the CASR-SF for both depression (OR 2.17, 95% CI 1.71–2.66) and anxiety (OR 2.16, 95% CI 1.72–2.60). When breaking down the CASR-SF by subscale, all types of violence (physical, psychological and sexual) showed an association with worsening depression and anxiety symptoms. The strongest association out of the three was seen for sexual violence (depression OR 2.12, 95% CI 1.47–2.78; anxiety OR 2.22, 95% CI 1.55–2.90). Sexual violence as measured through the CASR-SF was the strongest predictor of symptoms of anxiety out of all types of violence measured across all scales. Physical violence showed the weakest association with symptoms of depression and psychological violence showed the weakest association with symptoms of anxiety, however, women who experienced these types of violence still had almost 2 times the odds of poorer mental health than women who did not experience these types of violence (Figure 5; Table 3, CASR-SF).

Acts of technology-facilitated abuse (CARS-variables) were also associated with more severe symptoms of depression and anxiety. The global score for the CARS scale showed that women who experienced any kind of technology-facilitated abuse had around twice the odds of having more severe depressive and anxiety symptoms than women who did not experience technology-facilitated abuse. When looking at the subscales, psychological, sexual and stalking cyber abuse were all strongly associated with worsening depression and anxiety. The strongest association was seen for psychological cyber abuse, followed by stalking, and sexual cyber-abuse, in both cases (Figure 5; Table 3, CARS).

Experiences of economic violence (SEA2 variables) were also associated with more severe symptoms of depression and anxiety. Economic violence showed the strongest association with symptoms of depression out of all types of violence measured across all scales. Women who had experienced any economic violence, as represented by the global variable, had 2.21 times the odds of more severe depression symptoms than women who had not experienced economic violence (95% CI 1.82–2.69). Economic restriction and economic exploitation were similarly related to symptoms of depression (OR 2.16 vs. OR 2.14, respectively). Any experience of economic abuse was also related to worsening symptoms of anxiety. The subscales were stronger predictors of anxiety than the global variable, with economic restriction showing the strongest association (OR 2.00, 95% CI 1.60–2.42; Figure 5; Table 3, SEA2).

Polyvictimisation was a predictor of worse depression and anxiety, with the greater the number of different types of violence experienced by women, the greater the odds of having more severe symptoms. The odds of having more severe symptoms of depression were significantly increased for women who experienced all eight different types of violence compared to women who experienced just one type (OR 3.97, 95% CI 1.76–7.47). The same pattern was seen for anxiety, with an increase in the odds of more severe anxiety symptoms for women who experienced all types of violence compared with women who experienced just one (OR 3.75, 95% CI 1.63–7.66; Figure 6; Table 4, Num. of violence type).

Finally, the number of different distinct acts of violence that women experienced was also associated with increased symptoms of depression and anxiety. Women who experienced more distinct acts of violence were more likely to have more severe symptoms of depression and anxiety compared with women who experienced fewer acts of violence (Figure 6; Table 4, Num. of violence acts).

## 4 Discussion

In this study we explored the experiences of different types of IPV, including physical, psychological, sexual, economic and cyber IPV faced by migrant women living in the Valencian Community of Spain and the associations with symptoms of depression and anxiety. At almost 60%, the prevalence of any experience of violence in this study is much higher than in other studies on IPV. A recent systematic review and meta-analysis of global IPV studies found a pooled prevalence of any IPV in the past year of 24% (4), and the global prevalence of any lifetime IPV has been estimated at around 30–40% (4, 79). A study of around 10,000 women attending primary care centres in Spain found a lifetime prevalence of IPV of almost 25% (45). The increased prevalence in our study could reflect the use of a convenience sample, and that women who wanted to share their experiences may have been more motivated to participate. However, it could also be due to the fact that more types and acts of IPV were assessed, compared to other studies that have only assessed physical, psychological and sometimes sexual violence. Assessing more nuanced experiences of IPV increases the chances that women may report a specific act of violence, particularly for some of the more common acts of economic or technology-facilitated abuse such as a partner keeping financial information hidden, checking phones without permission, or intentionally ignoring text messages or phone calls to hurt feelings. It could also support the existing literature, which suggests that migrant women are at increased risk of IPV (31, 32). Studies from Spain have found that migrant women are at increased risk of experiencing IPV compared with Spanish-born women (45–47), however the prevalence of IPV in our study was still higher than in other studies of migrant women in Spain (47, 80).

Reviews of global IPV studies have found that psychological violence is often the most prevalent form of IPV experienced (4, 81), however our study found that economic violence was the most commonly reported form of violence. Given that most studies to date have not assessed the prevalence of economic violence alongside the prevalence of more frequently studied forms of IPV, the importance of economic violence in patterns of IPV may have been overlooked (9). The second most prevalent form of violence was technology-facilitated psychological abuse, which reflects the growing influence of technology within intimate relationships and IPV perpetration patterns (15, 20).

Around 70% of women reported some symptoms of depression and/or anxiety in the past 2 weeks, with 10%–15% of women reporting severe symptoms. This is in line with other studies that have shown migrant women are at increased risk of mental health sequelae (37, 82). The finding that women who had lived in Spain for a longer amount of time had poorer mental health than women who had been in Spain for less time is supported by other studies that have found that living conditions for migrants in the destination country, including discrimination, acculturation difficulties, and lack of access to good housing for example, can increase social suffering as time progresses, leading to a greater impact on mental health (33, 35). Previous research has also documented the impact of higher income level and the presence of social support in the protection against poor mental health in migrant groups (34, 35), supporting the findings in our study. Finally, women from Latin America and from Africa, Asia or the Middle East were more likely to experience worse symptoms of depression and anxiety than women from Europe or North America/Other. This could tie in with the literature on migrant experiences in destination countries which suggests that integration and acculturation can be impacted by cultural differences, as well as the ongoing presence of interpersonal and institutional discrimination (34, 35). Women who were not religious being more likely to experience worse symptoms of depression than women who were religious could reflect previous research that has shown women often use religion as a coping mechanism to protect against poor mental health. Not only are religious communities and leaders places that offer social support and advice to women, but women have reported in qualitative studies that praying or believing that their current situation is part of a Higher Plan for them helps them to cope with negative experiences (83, 84).

All types of violence showed a strong association with symptoms of depression. Economic violence was the strongest predictor, followed by sexual violence and psychological violence. Technology-facilitated abuse had almost the same impact on depression as abuse perpetrated in person. This reflects findings from previous studies that showed that cyber IPV was as detrimental to mental health as face-to-face IPV (20). The mental health impacts of technology-facilitated abuse may also be exacerbated by the fact that some of the acts of this type of abuse simultaneously prevent women from seeking help either from professional sources or from family or friends due to the surveillance of devices (17). This reiterates the notion that technology-facilitated abuse is a serious form of IPV which can have equally detrimental impacts on survivors. As technologies continue to develop, it is likely that new ways of perpetrating violence will emerge, for example one study describes how artificial intelligence and the use of deep fakes are now being used to perpetrate IPV (85). It is therefore imperative that technology-facilitated abuse be given equal attention in research, policy and programming spaces.

The strength of the relationship between economic abuse and depression could reflect the idea that economic abuse requires the least proximity to the victim in order to perpetrate. For example, it does not require physical proximity or open lines of communication to perpetrate, and can therefore continue for the longest amount of time compared to other forms of abuse, particularly after a relationship has ended (10). Economic abuse can therefore have longer lasting consequences, even once it has ended, that go beyond the immediate psychological and physical impacts of other forms of IPV. The use of economic violence to establish economic dependency on the partner whilst in the relationship, which impacts on their ability to leave the abusive situation and be financially independent once a relationship has ended, plus the long lasting impacts of economic abuse on women’s lives (10, 86) could increase and prolong the stress women face. Women have reported in previous studies that economic and financial difficulties are one of the main barriers to leaving an abusive partner (9). This increased stress could therefore increase their risk of depression, with chronic stress often predicting poorer depression outcomes (87). Supporting the findings from this study, other research has shown that women who experienced economic abuse were more likely to have symptoms of depression than women who did not experience economic abuse, and that economic abuse had a stronger association with symptoms of depression than other forms of non-physical abuse such as psychological abuse (10, 28).

The relationship between economic violence and depression is likely to have been exacerbated by women’s migrant status which makes them more vulnerable to economic insecurity, and social isolation, particularly when they have difficulties gaining employment or face downward mobility in their work (35). A previous study of IPV in migrant women in Spain found that having paid work was not a protective factor against IPV, which the authors suggested was due to the challenges that migrant women face when trying to enter the labor market (47). Migrant men also face these challenges (33, 35), which have been shown to increase the risk of IPV (88). Previous studies in Spain have demonstrated that higher levels of male unemployment and income inequality between men and women increase women’s risk of IPV (89), and that lower family income level is associated with an increased risk of IPV in migrant women living in Spain (47). Alternatively, women may be more isolated and dependent on their partners financially due to their migrant status (90). In this context, where women are already more vulnerable economically, acts of abuse linked to money or to economic factors may have a greater impact on women’s mental health. Studies have shown that financial independence is protective against poor mental health (91), therefore a lack of financial independence due economic abuse, which also prevents women from leaving an abusive situation, and exacerbated by migrant status is likely to be considerably detrimental for mental health.

Our findings showed that all types of violence also had a strong association with symptoms of anxiety. The strongest association was seen for sexual violence, followed by physical and psychological. This is supported by some previous research (4, 23), but is in contrast to other studies that have shown psychological abuse to be the strongest predictor of poor mental health, including anxiety (24–26). There have been limited studies that have reported on the relationship between sexual IPV and anxiety specifically, however a recent meta-analysis found that there was a stronger relationship between anxiety and sexual and psychological violence than there was between anxiety and physical violence. The authors suggested that this could be linked to attachment theory and that individuals with anxious attachment styles look for emotional regulation via their partner, may not recognise the warning signs of violence, and may have difficulties ending the abusive relationship due to an ‘intense fear of abandonment’ (92). A cohort study in Australia found that whilst IPV was not a predictor of new onset anxiety disorders, it was a strong predictor of anxiety in women with a previous diagnosis (93), therefore women who suffer with anxiety may be at increased risk of worsening symptoms after having experienced IPV. This may be exacerbated for migrant women who face an increased risk of social isolation and community ostracisation when attempting to disclose violence or leave an abusive relationship (32). Further research is warranted in this area to understand the temporal relationship and ongoing impacts of sexual IPV on anxiety symptoms.

Polyvictimisation, with multiple forms of violence experienced, and multiple distinct acts of violence were the strongest predictors of symptoms of depression and anxiety in our study. This is supported by other recent studies which have shown that experiencing multiple forms of IPV can increase survivors’ vulnerability to health sequelae such as headaches, chronic pain and sleep difficulties (94), as well as poor mental health (95, 96). Cantu and colleagues found that when looking at cyber IPV, there was an additive effect of multiple types of cyber abuse on symptoms of depression, with people who experienced all three types of cyber abuse having a 2.15 unit increase in depression score after controlling for face-to-face IPV (20). The presence of technology-facilitated abuse may also lead to face-to-face abuse, adding to patterns of polyvictimisation. For example, a qualitative study of online domestic abuse forums found that perpetrator monitoring and surveillance would often escalate into physical violence when the perpetrators discovered pieces of information that they did not like, as a result of their surveillance (17).

There is a high correlation between different forms of violence, which creates an intricate and complex pattern of experiences. For example, experiences of economic violence have been shown to be associated with an increase in experiences of psychological, physical and sexual violence (10). As the number of different acts or types of violence increases, it fits that their impact compounds, so that the more different types of violence a woman experiences, the worse her mental health is. In a study on dating violence in US college students, Sabina and Straus (72) were some of the first to look at the impact of polyvictimization within intimate relationships on mental health and they found that the co-existence of psychological, physical and sexual violence was the strongest predictor of post-traumatic stress and depression symptoms in women. Our findings therefore add support to the notion of complex IPV patterns and that experiences compound to have worsening impacts on women’s mental health.

### 4.1 Strengths and limitations

Our study makes a novel contribution to the literature in a number of ways. To our knowledge, this is the first study to assess economic abuse and technology-facilitated abuse alongside more traditionally studied forms of IPV, to assess the differential relationship of IPV with depression and anxiety disaggregated by each type of IPV and to assess the relationship between type of IPV and mental health in migrant groups in Spain. These findings can help to better understand the patterns of IPV experiences faced by migrant women, and their mental health impacts, in order to plan for effective policy implementation and service provision.

Research with hard-to-reach populations inevitably misses the most vulnerable of participants, however, and our study is no different. The use of non-probabilistic sampling methods and the use of social media to advertise the survey risks excluding women who are not well connected through these means, who have limited access to technology or who are extremely socially isolated. The majority of respondents were from European countries, were working, and had adequate salaries. Whilst this reflects the general migrant population in Spain (43), it may have missed certain groups of migrant women who are at further increased risk of IPV and poor mental health, which makes the generalisability of findings more difficult. In addition, although the survey was administered in multiple languages in order to capture as many women as possible, it may have excluded women who do not speak Spanish, English or French.

The use of cross-sectional surveys prevents the assessment of causality in the relationship between IPV and mental health. In addition, a variety of nuance and detail may have been lost in the management of the variables necessary for this analysis. In order to reduce the risk of small numbers and complete separation, socio-demographic categories, particularly for country of origin were collapsed and summarised. This prevents a more nuanced understanding of how the relationship between violence and mental health impacts women from different ethnic, cultural, racial and linguistic backgrounds, which is important for service planning. Future studies with larger sample sizes may be able to explore this nuance further.

The nature of IPV perpetration patterns is ever changing, particularly with the rapid invention of new technologies and the associated technology-facilitated IPV that may result. Whilst there are still limited studies on technology-facilitated abuse and our research makes an important contribution to this space, the tool we used to assess cyber IPV was developed in 2016. It is likely that new ways of perpetrating IPV via technology have since emerged and it is possible that some of this detail was missed. This will be an important area of study in the future as technology continues to evolve at a fast pace, and particularly with the recent availability of Artificial Intelligence to the general public.

Future studies may want to consider the use of more probabilistic sampling methods directed at hard-to-reach populations, as well as cohort studies to assess the directionality of the relationship between different types of IPV and mental health. Whilst our study began to look at the differential association of types of IPV with mental health, including economic and technology-facilitated abuse, further research may want to add to this literature base. In addition, future research may want to assess the severity of violence, accounting for the range of different types of violence experienced, as well as their frequency, to come to a more comprehensive understanding of the impact of different violence experiences on mental health.

## 5 Conclusion

In line with our objectives, this study investigated the association between different forms of IPV and symptoms of anxiety and depression in migrant women in Spain in order to explore the most detrimental forms of violence for migrant women’s mental health. Our findings show that all forms of IPV are associated with poor mental health, but economic and sexual IPV are of particular concern. IPV consists of complex perpetration patterns of polyvictimisation that can compound to worsen migrant women’s mental health, and newer and under-studied forms of violence such as technology-facilitated abuse also demonstrate important associations with mental health that should not be overlooked in research and service provision. The findings from this study can be used by service providers and policy makers to advocate for wider and more comprehensive definitions of IPV in order to identify women at risk and facilitate access to support. Additionally, they should be used to demonstrate the need for accessible and appropriate mental health services for migrant women and other marginalised survivors of IPV who face multiple and intersecting risks to their mental health.

## Data availability statement

The datasets presented in this study can be found in online repositories. The names of the repository/repositories and accession number(s) can be found at: Zenodo, https://doi.org/10.5281/zenodo.8409602.

## Ethics statement

The studies involving humans were approved by Ethics Committee of Research in Humans, University of Valencia. The studies were conducted in accordance with the local legislation and institutional requirements. The participants provided their written informed consent to participate in this study.

## Author contributions

AB: Conceptualization, Data curation, Formal Analysis, Funding acquisition, Investigation, Methodology, Project administration, Writing – original draft, Writing – review & editing. GR-M: Formal Analysis, Methodology, Software, Writing – original draft, Writing – review & editing.
